# Severe 3,4-Methylenedioxymethamphetamine (MDMA)-Associated Rhabdomyolysis in Crohn’s Disease: Direct Toxicity and Inflammatory Susceptibility in the Absence of Hyperthermia

**DOI:** 10.7759/cureus.101526

**Published:** 2026-01-14

**Authors:** Sebastian Hernandez Mejia, Aishwarya Ashwinee, Ranwa Aldaker

**Affiliations:** 1 Internal Medicine/Cardiology, Richmond University Medical Center, New York, USA; 2 Internal Medicine, Richmond University Medical Center, New York, USA; 3 Internal Medicine, St. George's University School of Medicine, St. George's, GRD

**Keywords:** 4-methylenedioxymethamphetamine (mdma), acute kidney injury, chron's disease, inflammatory bowel disease, inflammatory myopathy, mdma toxicity, mitochondrial dysfunction, recreational drug use

## Abstract

Rhabdomyolysis is a recognized complication of 3,4-methylenedioxymethamphetamine (MDMA), usually driven by severe hyperthermia and agitation. We describe a previously healthy 45-year-old man who developed profound rhabdomyolysis (creatine kinase (CK) 160,000 U/L) and intrinsic acute kidney injury requiring hemodialysis after ingesting 1.5 g of “Molly” (pure MDMA), despite remaining afebrile throughout. In the literature review, the first reported case of severe MDMA-associated rhabdomyolysis without hyperthermia and the third-highest CK values was documented. He later received a new diagnosis of Crohn’s disease, raising the possibility that inflammatory bowel disease-related myositis created an “immune-primed” susceptibility to muscle injury. In contrast to typical MDMA cases, there was no hyperthermia, exertion, serotonin syndrome, or significant electrolyte abnormality to explain the muscle breakdown. This case, therefore, supports an under-recognized, non-hyperthermic mechanism of MDMA toxicity involving direct mitochondrial and oxidative skeletal muscle injury, potentially amplified by occult Crohn’s disease, and highlights the need to consider MDMA toxicity even in afebrile patients presenting with severe rhabdomyolysis.

## Introduction

3,4-Methylenedioxymethamphetamine (MDMA), or ecstasy, is a popular party drug among young adults. Contaminated tablets are known to cause serious adverse and toxic effects. However, Molly, a “clean and pure” form of MDMA, is often seen as a safer alternative. Notable among the rare effects experienced by those who ingest pure MDMA is rhabdomyolysis associated with hyperthermia [1-4].

In most reported cases with acute MDMA toxicity-induced rhabdomyolysis, serum creatine kinase (CK) levels peak between 250 and 10,000 U/L, with CK levels >10,000 U/L only seen in a small minority (2.4%). More relevant, the majority of cases associate the syndrome of hyperthermia with rhabdomyolysis [5-7].

We report a previously healthy man who developed extreme rhabdomyolysis (CK 160,000 U/L), the third-highest CK values documented after first-time ingestion of Molly, despite remaining entirely normothermic. Follow-up evaluation revealed Crohn’s disease, echoing rare reports where disproportionate rhabdomyolysis was later attributed to inflammatory bowel disease-related myositis, suggesting that chronic intestinal inflammation may have amplified MDMA-induced muscle injury. Based on our review, this appears to be the first reported case of severe MDMA-associated rhabdomyolysis without hyperthermia. Importantly, this case highlights MDMA’s potential for direct mitochondrial and oxidative skeletal muscle toxicity independent of hyperthermia, an under-recognized mechanism with minimal clinical investigation.

## Case presentation

A previously healthy 45-year-old man complained of acute generalized weakness, severe myalgias, and inability to ambulate after first-time ingestion of 1.5 g of Molly, a pure form of MDMA. Symptoms started acutely after the third dose of 0.5 g, for a total of 1.5 g over 10 hours while at home. On admission to the emergency department, the patient was awake, markedly agitated, and paranoid, unable to move because of diffuse muscle pain, rigidity, and stiffness, with no signs of apparent trauma, convulsive seizures, or strenuous physical exertion. Core temperature (rectally) ranged from 97.1 to 98.1°F with a Glasgow Coma Scale of 14. The only positive examination findings were tachycardia, hyperreflexia, and tea-colored urine, which gave the clue to suspect possible rhabdomyolysis.

**Figure 1:**
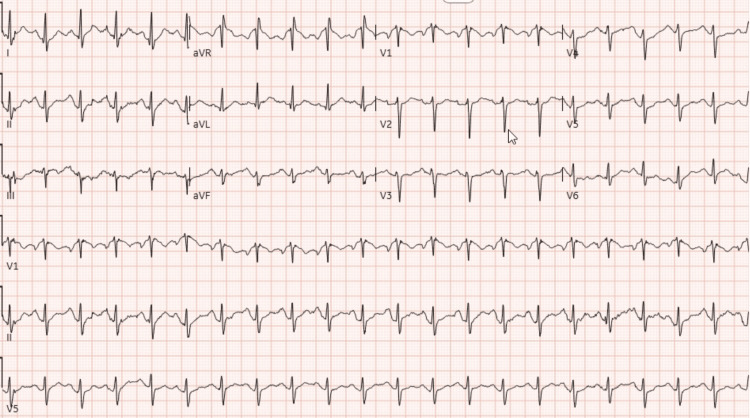
Electrocardiogram on admission Sinus tachycardia with normal QRS complexes and no ST-segment or T-wave changes suggestive of hyperkalemia or ischemia. Baseline tremor artifact is consistent with muscle rigidity in the setting of MDMA toxicity. MDMA: 3,4-methylenedioxymethamphetamine

Initial management for agitation was rapidly achieved with intravenous benzodiazepines. At the time, laboratory workup was confirmatory for severe rhabdomyolysis; the initial CK level was close to 120,000 u/L and peaked at 160,000 u/L 24 hours after the last MDMA dose. Additional compatible bloodwork was a positive urinalysis for myoglobinuria, acute kidney injury with a fractional excretion of sodium of 5.8% consistent with intrinsic kidney injury, anion gap metabolic acidosis, and elevated liver enzymes. An electrocardiogram is remarkable for sinus tachycardia, normal segments and intervals, and negative for any signs of hyperkalemia (Figure 1). The urine toxicology showed benzodiazepines, but no amphetamines or other drugs were found.

**Figure 2:**
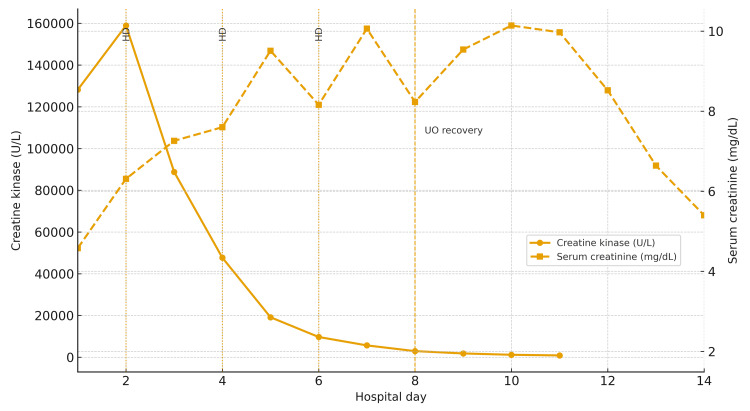
Trend of CK and serum creatinine during hospitalization CK (solid line) peaked at 159,952 U/L on day 2 and then declined, while serum creatinine (dashed line) rose to 10.14 mg/dL by day 10 before improving. Dotted lines mark hemodialysis days (2, 4, and 6), and the dashed line at day 8 indicates recovery of urine output. CK: creatine kinase

Due to the rapid rise in serum creatinine and severe acidemia, urgent hemodialysis was warranted within the first 12 hours of presentation, and the patient was maintained on telemetry for close observation. Notably, there was no clinical or laboratory evidence of disseminated intravascular coagulation, compartment syndrome, or acute hepatic failure. The total length of stay was 14 days; he was treated with intravenous fluids to maintain a goal urine output of at least 1 to 2 mL/kg/h, which he could not achieve the first week, producing only around 100 mL in 24 hours (0.05 mL/kg/h). Pertinently, the desired urine output was achieved on the eighth day; as a result, the decision to discontinue hemodialysis after three sessions, on the second, fourth, and sixth days, was made. His creatinine peaked at 10.14 mg/dL on the 10th day, followed by a satisfactory, steady decline (Figure 2).

In a one-month follow-up post-discharge, his general, physical, and systemic examinations were within normal limits, creatinine levels had nearly normalized, and serum myopathy workup was negative. However, a persistently elevated C-reactive protein together with positive anti-*Saccharomyces cerevisiae* IgG/IgA antibodies prompted evaluation for underlying inflammatory bowel disease, and a colonoscopy subsequently confirmed a diagnosis of Crohn’s disease.

## Discussion

MDMA is a commonly used recreational drug whose acute toxicity is well described, including hyperthermia, hyponatremia, serotonin syndrome, rhabdomyolysis, and acute kidney injury [1-4]. Large series such as the Euro-DEN registry describe rhabdomyolysis in a minority of acute recreational drug presentations. Still, when it occurs in the setting of MDMA, it is usually linked to severe hyperthermia and prolonged agitation or exertion [1,2,4]. In most published MDMA cases, CK peaks between about 30,000 and 100,000 U/L, with only a few reports exceeding this range [1,2,5,6]. Our patient’s peak CK of 160,000 U/L places him among the highest CK values reported in MDMA-associated rhabdomyolysis, with only two cases in the literature clearly documenting higher levels [5,6].

MDMA triggers a rapid release of serotonin and norepinephrine from nerve terminals, producing its characteristic sympathomimetic and neuropsychiatric effects: tachycardia, hypertension, agitation, increased muscle tension, and heightened arousal [3,4,7-10]. MDMA also activates key serotonergic receptors in the hypothalamus, particularly 5-HT2A and 5-HT1A, which further influence thermoregulation, sensory processing, and mood [8,10,11]. These mechanisms underlie the well-known subjective effects of increased energy, emotional openness, and euphoria, while increasing heat production through enhanced muscle activity and simultaneously impairing the body’s ability to dissipate heat [7,11].

At higher doses, hepatic metabolic pathways become saturated, leading to accumulation of reactive metabolites such as MDA and α-methyldopamine (α-MeDA), which generate reactive oxygen species and disrupt intracellular calcium homeostasis [12-15]. These oxidative and calcium-mediated stresses promote mitochondrial uncoupling in skeletal muscle by activating uncoupling proteins and destabilizing the inner mitochondrial membrane. The resulting loss of the proton gradient diverts oxidative phosphorylation away from adenosine triphosphate (ATP) synthesis and forces excess substrate oxidation, releasing energy as heat and compounding mitochondrial dysfunction [12-15]. Together, these disturbances create an energy-deficient, highly injury-prone muscular environment that predisposes to extensive myocyte breakdown.

Taken together, these mechanisms explain why the most severe presentations of MDMA toxicity in the literature have overwhelmingly been attributed to hyperthermia-driven muscle injury, as dysregulated central thermoregulation-often intensified by environmental heat or prolonged exertion-can raise core temperatures above 40°C. Experimental animal work has shown that elevated ambient temperatures markedly exacerbate MDMA-induced hyperthermia and neurotoxicity [16]. In animal models, MDMA disrupts autonomic thermoregulation [17] and induces mitochondrial uncoupling in skeletal muscle [18], both of which directly increase heat production and promote rhabdomyolysis. Consistently, human case reports of fatal MDMA intoxication almost uniformly document core temperatures exceeding 40°C [19]. At these extreme temperatures, heat accelerates mitochondrial dysfunction, ATP depletion, intracellular calcium overload, myofibrillar breakdown, coagulation abnormalities, and ultimately multi-organ failure [20]. This hyperthermic cascade accounts for the majority of MDMA-associated rhabdomyolysis cases reported to date [1,2,4].

The most striking feature of this case is not only the severity of the rhabdomyolysis, but the fact that it occurred without any of the physiologic triggers that traditionally explain MDMA-related muscle injury. With hyperthermia, exertion, serotonin syndrome, and electrolyte derangements all absent, the clinical picture forced us to consider alternative, non-thermogenic pathways.

Our research points toward MDMA causing direct muscle toxicity through mitochondrial and oxidative injury, independent of hyperthermia, a pathway rarely emphasized but well supported by experimental evidence [21]. After ingestion, MDMA is rapidly O-demethylenated to MDA and subsequently oxidized to α-MeDA, catechol metabolites that readily conjugate with glutathione or cysteine. Although conjugation with glutathione or cysteine is typically a detoxification pathway, in this setting, it yields even more reactive and toxic metabolites, forming ortho-quinone thioether conjugates. These thioether species undergo continuous redox cycling, generating large amounts of reactive oxygen species, which drive lipid peroxidation and rapidly deplete intracellular antioxidant reserves, particularly glutathione, resulting in profound oxidative stress [22-24].

This oxidative injury directly targets mitochondria: thioether metabolites destabilize the mitochondrial membrane potential, impair electron transport at complexes I and III, and reduce ATP synthesis, creating an immediate state of cellular energy failure [22-24]. Significantly, recent in vivo evidence further strengthens this mechanism. In a rodent model of MDMA-induced nephrotoxicity, Roghani et al. demonstrated time-dependent mitochondrial dysfunction, increased lipid peroxidation, depletion of antioxidant reserves, and activation of caspase-mediated apoptosis in renal tissue after MDMA exposure, providing compelling evidence that MDMA induces mitochondrial toxicity in peripheral organs, not only in the CNS [25]. Unlike the hyperthermia pathway, where mitochondrial failure is secondary to extreme temperature, here the mitochondrial dysfunction is primary, with oxidative stress itself initiating the collapse of mitochondrial energetics.

Perhaps the most striking aspect of this case is the patient’s newly diagnosed Crohn’s disease, uncovered on follow-up colonoscopy after discharge. Although rare, inflammatory bowel disease has been linked to episodes of disproportionate rhabdomyolysis through inflammatory myositis [26]. A small number of published reports describe patients with Crohn’s disease developing very high CK levels after minimal exertion or even at rest, where further evaluation revealed muscle inflammation as the underlying process. Matsuda et al. reported recurrent rhabdomyolysis in a Crohn’s patient later found to have myositis, initially mistaken for drug-induced injury [27]. Chiba et al. described an early case of Crohn’s-associated rhabdomyolysis without hyperthermia or exertion, suggesting an immune-mediated mechanism [28]. Hayashi et al. documented markedly elevated CK with biopsy-proven myositis even in the absence of muscle symptoms, showing how subtle this complication can be [29]. More recently, Tran et al. reported a young man who experienced CK >55,000 U/L after light exercise and was subsequently diagnosed with Crohn’s disease, supporting the idea that muscle involvement can precede GI symptoms [30].

Taken together, these rare but informative reports suggest that Crohn’s disease can create a muscle environment that is immunologically “primed” and unusually vulnerable to injury, even before classical GI symptoms are recognized. Within this framework, our patient may have had an unrecognized inflammatory susceptibility in his skeletal muscle. High-dose MDMA, with its known mitochondrial toxicity, oxidative stress, and impaired calcium handling, may then have acted as the final trigger, dramatically amplifying muscle damage despite the absence of hyperthermia or exertion. This dual-hit model (inflammatory bowel disease-related immune vulnerability plus MDMA-induced metabolic stress) provides a clear and biologically plausible explanation for the unusually severe rhabdomyolysis observed in this case.

Lastly, this case highlights the renal impact of profound rhabdomyolysis in MDMA toxicity and the importance of rapid intervention. In our patient, the extremely elevated CK level and severe metabolic acidosis were most consistent with pigment-induced intrinsic kidney injury, a well-recognized consequence of massive myoglobin release. Despite the severity of his presentation, early aggressive hydration and prompt initiation of hemodialysis led to full renal recovery. This emphasizes that even without hyperthermia, MDMA-related rhabdomyolysis can cause severe but reversible acute kidney injury when clinicians act quickly and escalate care without delay.

## Conclusions

This case prompts a re-evaluation of our current understanding of MDMA-associated rhabdomyolysis in the absence of hyperthermia, an area with very limited clinical investigation. By integrating prior and newer experimental and clinical work, it supports a broader view of MDMA toxicity in which severe injury results not only from hyperthermia but also from time-dependent direct mitochondrial and oxidative damage, a mechanism shown in experimental models to affect multiple organs, including skeletal muscle, cardiomyocytes, neuronal tissue, and kidneys. In addition, the rare association of Crohn’s disease with myositis and markedly elevated CK despite minimal or no physiologic stress suggests that IBD-related immune activation may make skeletal muscle unusually susceptible to disproportionate injury, even when classical physiologic triggers are absent. Our patient’s full renal recovery after early aggressive hydration and timely hemodialysis also shows that severe pigment-induced acute kidney injury in this setting can remain reversible when recognized and treated promptly. It highlights the need for further clinical research into these non-hyperthermic mechanisms of MDMA toxicity.
